# SAS score: Targeting high-specificity for efficient population-wide monitoring of obstructive sleep apnea

**DOI:** 10.1371/journal.pone.0202042

**Published:** 2018-09-05

**Authors:** Alexandru Topîrceanu, Mihai Udrescu, Lucreţia Udrescu, Carmen Ardelean, Rodica Dan, Daniela Reisz, Stefan Mihaicuta

**Affiliations:** 1 Department of Computer and Information Technology, Politehnica University of Timişoara, Timişoara, Romania; 2 Faculty of Pharmacy, “Victor Babeş” University of Medicine and Pharmacy Timişoara, Timişoara, Romania; 3 Department of Pulmonology, “Victor Babeş” University of Medicine and Pharmacy Timişoara, Timişoara, Romania; 4 Department of Cardiology, “Victor Babeş” University of Medicine and Pharmacy Timişoara, Timişoara, Romania; 5 Department of Neurology, “Victor Babeş” University of Medicine and Pharmacy Timişoara, Timişoara, Romania; 6 Timişoara Institute of Complex Systems, Timişoara, Romania; University of Rome Tor Vergata, ITALY

## Abstract

**Proposal:**

This paper investigates a novel screening tool for Obstructive Sleep Apnea Syndrome (OSAS), which aims at efficient population-wide monitoring. To this end, we introduce *SAS*_*score*_ which provides better OSAS prediction specificity while maintaining a high sensitivity.

**Methods:**

We process a cohort of 2595 patients from 4 sleep laboratories in Western Romania, by recording over 100 sleep, breathing, and anthropometric measurements per patient; using this data, we compare our *SAS*_*score*_ with state of the art scores STOP-Bang and NoSAS through area under curve (AUC), sensitivity, specificity, negative predictive value (NPV), and positive predictive value (PPV). We also evaluate the performance of *SAS*_*score*_ by considering different Apnea–Hypopnea Index (AHI) diagnosis cut-off points and show that custom refinements are possible by changing the score’s threshold.

**Results:**

*SAS*_*score*_ takes decimal values within the interval (2, 7) and varies linearly with AHI; it is based on standardized measures for BMI, neck circumference, systolic blood pressure and Epworth score. By applying the STOP-Bang and NoSAS questionnaires, as well as the *SAS*_*score*_ on the patient cohort, we respectively obtain the AUC values of 0.69 (95% CI 0.66-0.73, *p* < 0.001), 0.66 (95% CI 0.63-0.68, *p* < 0.001), and 0.73 (95% CI 0.71-0.75, *p* < 0.001), with sensitivities values of 0.968, 0.901, 0.829, and specificity values of 0.149, 0.294, 0.359, respectively. Additionally, we cross-validate our score with a second independent cohort of 231 patients confirming the high specificity and good sensitivity of our score. When raising *SAS*_*score*_’s diagnosis cut-off point from 3 to 3.7, both sensitivity and specificity become roughly 0.6.

**Conclusions:**

In comparison with the existing scores, *SAS*_*score*_ is a more appropriate screening tool for monitoring large populations, due to its improved specificity. Our score can be tailored to increase either sensitivity or specificity, while balancing the AUC value.

## Introduction

Obstructive sleep apnea syndrome (OSAS) is a serious sleep respiratory disorder, which has a prevalence that is considered by many authors as epidemic [1–6]. OSAS consists of abnormal breathing pauses that occur during sleep, resulting in sleep fragmentation and excessive daytime somnolence [7, 8]; it is considered as part of the wider category named SDB (sleep-disordered breathing). In general, SDB produces an impaired quality of life, including an increased risk of causing motor-vehicle accidents. SDB also increases the mortality rate [9], because it contributes to the development of cardiovascular diseases [10] such as hypertension [11], type 2 diabetes [12], cancer [13], and chronic kidney disease [14]. Because it is associated with many co-morbidities [15], SDB has several distinct clinical phenotypes. If not properly diagnosed and treated, SDB may increase morbidity and preoperative risks as well [16–20].

OSAS severity is quantified with the Apnea-Hypopnea Index (*AHI*). Apneas are defined as a decrease of at least 90% of airflow from baseline, which lasts for ≥ 10 seconds, whereas hypopneas are defined as a ≥ 30% decrease of airflow that lasts ≥ 10 seconds; both are associated with either an arousal or a ≥ 3% *O*_2_ saturation decrease [21]. The AHI represents the mean number of apneas and hypopnoeas per hour of sleep. Clinically significant OSAS is characterized by AHI ≥ 30. However, some studies are adopting different AHI thresholds for OSAS, such as 15 (considered as the lower limit for moderate risk) or 20 [22]. Nonetheless, the clinical relevance and consequences of mild obstructive sleep apnoea is still unclear [23]. Also, there is a variability in scoring the respiratory events across different countries [24].

In current practice, there are three major predictive scores based on questionnaires, namely Berlin (since 1999), STOP-Bang (since 2008), and NoSAS score (since 2016) [22, 25–28]. STOP-Bang is considered as the better alternative to Berlin score, due to its high sensitivity rates (83-100%). STOP-Bang has a low specificity (37-56%) [27, 28], which prevents its usage for large population screening. NoSAS score comes to improve the prediction specificity by a considerable margin (69%), while maintaining a sufficient sensitivity value (79%).

Present practice shows that the existing screening tools are limited when monitoring large populations (e.g. groups of more than 100, 000 people). In other words, current scores mainly focus on simplicity and high sensitivity, because these characteristics are paramount for clinical problems such as a rapid diagnosis of preoperative patients—the unfortunate consequence is a high rate of false positives.

This paper builds upon our previous work to improve the *SAS*_*score*_ [29] by specifically aiming at a high specificity. At the same time, to address the needs of practitioners in sleep laboratories, we simplify the computation of the original score, so that it may easily be applied in daily scenarios. Altogether, the contributions of this paper are:

- We redefine our computer-based algorithm that calculates *SAS*_*score*_ in a form that can also be used by practitioners in a much simpler way, without the need to employ dedicated *in silico* tools. To this end, we only marginally reduce the accuracy of the original *SAS*_*score*_, while significantly increasing its usability.
- We validate the simplified *SAS*_*score*_ on a cohort of 2595 patients diagnosed in several sleep centers from Western Romania.
- We optimize the performance of our *SAS*_*score*_, to maximize its specificity (using AUC).
- We compare *SAS*_*score*_ with state of the art monitoring tools (i.e. STOP-Bang, NoSAS) in terms of sensitivity, specificity, AUC, to conclude that *SAS*_*score*_ is indeed better suited for monitoring large populations.

## Materials and methods

### Study design and participants

The study presented in this paper is based on the approval granted by The Ethical Committee of Victor Babes Hospital, Timisoara, Romania (approval no. 10/12.10.2013).

The “Western Romania” (*WestRo*, available as S1 Dataset) cohort consists of 2595 consecutive patients with suspicion of sleep breathing disorders, which were evaluated at several sleep laboratories in Timisoara (Western Romania) between March 2005 and May 2017. At the initial visit, the study protocol was clearly explained, to obtain the patient’s consent and the acceptance of referral physicians. Subsequently, cardiorespiratory polygraphy and polisomnography (PSG) were performed. Polygraphy was carried out with both Philips Respironics’ Stardust polygraph (2005) and SleepDoc Porti 7, while PSG was performed with Philips Respironics’ Alice 5 and Alice 6 Diagnostic Sleep System, according to the appropriate guidelines [30, 31]. The polygraphy was performed both at home and at the hospital, whereas PSG measurements were only performed under medical supervision (at the hospital). To preserve the information accuracy, all collected data were carefully verified. Throughout the entire process we ensured complete data confidentiality. Overall, our observational, retrospective study employs only standardized procedures that are non-invasive.

All 2595 patients with completed sleep study protocol and signed informed consent are included in the WestRo cohort, each with the corresponding 108 cardio-respiratory parameters and anthropometric measurements. Based on the collected data, we are able to compute not only the *SAS*_*score*_, but also state of the art scores STOP-Bang and NoSAS. For the STOP-Bang score we use the following parameters: gender (“M” = male), age (> 50 years), BMI (> 35 *kg*/*m*^2^), neck circumference (> 41/43 cm females/males), hypertension (“1” = yes), snoring (“1” = yes), sleepiness/tired (“1” = yes), observed apneas/agitated sleep (“1” = yes). To compute the NoSAS score we use the following parameters: gender (“M” = male), age (> 55 years), BMI (> 25/30 *kg*/*m*^2^), neck circumference (> 40 cm), snoring (“1” = yes). The parameter thresholds used to compute STOP-Bang and NoSAS have the exact values that were originally defined for each screening tool [27] [26] [22].

### State of the art screening tools for OSAS

To address the need for OSAS/SDB screening, state-of-the-art scores such as Berlin [25], STOPBang [27], and NoSAS [22] have been proposed.

The Berlin questionnaire includes information about snoring, daytime sleepiness and fatigue, obesity, and hypertension. It was developed by using a general clinical sample of 744 individuals, of whom 13% had their OSAS diagnosis confirmed through polygraphy [25].

The STOP-Bang score combines information from a self-administered questionnaire about complaints of snoring, tiredness, observed apnea, and high blood pressure, with clinical and anthropological parameters such as body mass index (BMI), age, neck circumference, and sex. It was created by processing a large group of 2477 patients that were assessed prior undergoing surgery. Out of these patients, 9% were diagnosed with OSAS [27].

Most literature recommends STOP-Bang over Berlin due to its higher sensitivity rates of 83.6% for *AHI* > 5, 92.9% for *AHI* > 15, and 100% for *AHI* > 30. However, STOP-Bang has a lower specificity (56.4% for *AHI* > 5, 43% for *AHI* > 15, and 37% for *AHI* > 30) [27, 28], which prevents its usage for large population screening. Although there are notable attempts to improve STOP-Bang’s specificity [32], they are mainly targeting narrow-type cohorts such as preoperative patients.

NoSAS is a relatively new score introduced by Marti-Soler et al. [22] that provides a good sensitivity for detecting individuals at risk of SDB. The score was developed based on multiple factor analysis and logistic regression to identify patients with clinically significant OSAS. The initial development of NoSAS was done on a cohort of 2121 participants from Lausanne (Switzerland); the result is a score between 0 and 17, which takes into consideration the following patient data: neck circumference (4 points if ≥ 40 cm), BMI (5 points if ≥ 30), snoring (2 points if present), age (4 points if ≥ 55 years), and gender (2 points if male). NoSAS is able to identify a significant risk of OSAS, if the score is bigger than the threshold value (i.e. ≥ 8).

NoSAS score was also applied on an Asian cohort, briefly after its publication in 2016, using a sample of 242 subjects from Singapore [33]. The same subjects were given the Berlin and STOP-Bang questionnaires before the study began. The results for predicting severe OSAS (defined as ≥ 30 events/h) indicate a sensitivity of 0.69 and a specificity of 0.73 for NoSAS. The AUC values were similar for all three questionnaires (within the interval 0.68-0.75). The authors conclude that NoSAS performed similarly to the STOP-Bang and Berlin questionnaires in a multi-ethnic Asian cohort [33], with no noticeable distinction in NPV or AUC. This study confirms that further improvements for OSAS prediction scores are required.

Overall, NoSAS is proven to have a good accuracy (AUC, sensitivity) compared to the other questionnaires (i.e. Berlin and STOP-Bang). Similarly to our proposed *SAS*_*score*_, it is estimated that NoSAS algorithm can be used for OSAS/SDB screening in larger cohorts where polysomnography is too expensive or time consuming.

### *SAS*_*score*_ development

To develop the original *SAS*_*score*_, we employed a two-step *Machine Learning* approach, as fully detailed in [29]. First, we performed dual clustering (*unsupervised learning*) on a complex network of OSAS patients using a relevant population of 1371 consecutive patients. In our complex network, nodes represent OSAS patients and links represent disease compatibility relationships that were defined according to a set of objective, easy-to-measure clinical and anthropometric parameters (their distributions are provided in S1 Table and S1 Fig). The processed network has 8 topological clusters, which we interpret as a set of 8 distinct OSAS-acquiring patterns (i.e. phenotypes). Then, we employed *supervised learning* to obtain a decision tree which assigns any new patient to one of the 8 discovered phenotypes. Subsequent statistical analysis is performed on each cluster/phenotype to render *SAS*_*score*_ according to each parameter’s cluster averages.

In this paper, we develop a new version of *SAS*_*score*_, in order to make it handy for clinical practitioners. As such, we propose a simplified method for computing *SAS*_*score*_ that does not require dedicated software tools, computers or smartphones. We also validate the new *SAS*_*score*_ on a cohort of 2595 patients from Western Romania, and provide a fair and consistent statistical comparison with state of the art questionnaires, by applying all scores/questionnaires on the same OSAS patients database.

Originally, *SAS*_*score*_ was created in such a way that, for every new patient, computer-based algorithmic processing is required to insert the patient into our curated apnea patient network. Then, the patient is automatically assigned to one of the 8 graph clusters (phenotypes); after performing this assignment, the patient’s *SAS*_*score*_ is computed with the following equation:

$$\begin{array}{c} SAS_{score}=\frac{BMI}{BMI_{cluster}}+\frac{NC}{NC_{cluster}}+\frac{SysBP}{SysBP_{cluster}}+\frac{ESS}{ESS_{cluster}} \end{array}$$ (1)

In Eq 1 the index of the assigned cluster is *cluster* ∈ {1..8}. Each cluster has a set of precomputed average measures: *BMI*_*cluster*_ (for body-mass index), *NC*_*cluster*_ (neck circumference), *SysBP*_*cluster*_ (systolic blood pressure), and *ESS*_*cluster*_ (Epworth Sleepiness Scale [34]). Thus, the new patient’s anthropometric and clinical parameters *BMI*, *NC*, *SysBP*, and *ESS* are normalized towards the cluster’s average values, so that his/her *SAS*_*score*_ represents a relative risk as compared to the cluster average. Such an approach is owing to the normal/Gaussian distribution that was identified in all relevant parameters and anthropometrics [29].

However, the computational steps entailed by calculating the original *SAS*_*score*_ require specialized, computer-based software tools. Therefore, while maintaining our initial focus on building a high specificity and sensitivity OSAS monitoring tool, we simplify Eq 1 according to the following principles:

- We take into consideration all relevant parameters that were rendered by the combined complex network and machine learning approach from [29]: *BMI*, *NC*, *SysBP*, and *ESS*.
- The difference from our original *SAS*_*score*_ from Eq 1 is that, instead of performing machine learning for cluster assignment followed by the dynamical adjustment of cluster-specific averages, we use fixed average values for the considered parameters.

Therefore, in Eq 2 the fixed average values for *BMI*, *NC*, *SysBP*, and *ESS* are standard values that can be found in literature and that are used in clinical practice. Over time, if other standard values will be embraced by clinicians and researchers, these fixed averages for *BMI*, *NC*, *SysBP*, and *ESS* can be updated. As a remark to Eq 2, the patient’s *NC* is divided by 40 cm for females (♀), or by 43 cm for males (♂).

$$\begin{array}{c} SAS_{score}=\{\begin{array}{cc} \frac{BMI}{30}+\frac{NC}{40}+\frac{SysBP}{140}+\frac{ESS}{11} & \text{,}♀\\ \frac{BMI}{30}+\frac{NC}{43}+\frac{SysBP}{140}+\frac{ESS}{11} & \text{,}♂ \end{array} \end{array}$$ (2)

**Fig 1:**
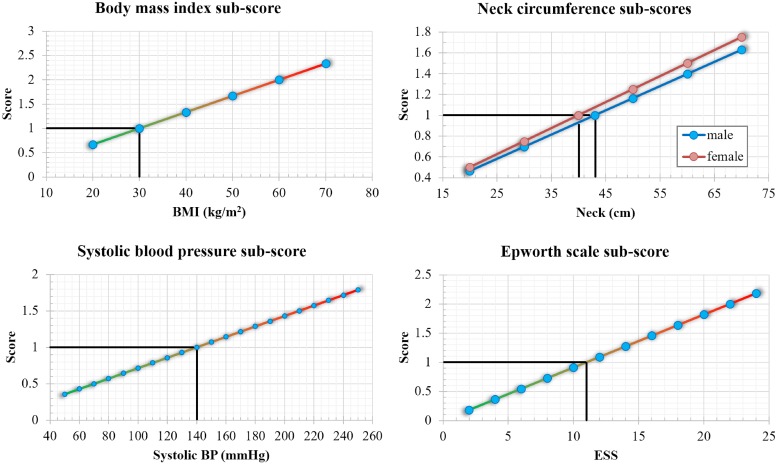
XY calculation plots for the sub-score of each of the four components within *SAS*_*score*_: $\frac{BMI}{30}$, $\frac{NC}{40}$ for females and $\frac{NC}{43}$ for males, $\frac{SysBP}{140}$, $\frac{ESS}{11}$. Each plot highlights with black lines the parameter value for which the respective sub-score equals 1.

With Eq 2, the computation of *SAS*_*score*_ on any new patient becomes a straightforward task. To provide an *offline* support for our method, any clinical practician, family doctor, or patient, may use the charts plotted in Fig 1.

The resulted score is a rational number with no strict lower or upper bound. Nevertheless, due to specific limits of anthropometric and physiological measures, we found that scores mainly range within the [2, 7] interval. Because the score is consistently proportional with the patient’s AHI, we also provide a direct risk classification which corresponds to the AHI-based risk groups:

$$\begin{array}{c} SAS_{Risk}=\{\begin{array}{ccc} Low & \text{if} & SAS_{score}<3\\ Mild & \text{if} & 3\leq SAS_{score}<3.5\\ Moderate & \text{if} & 3.5\leq SAS_{score}<4\\ High & \text{if} & 4\leq SAS_{score}<5\\ Very\;high & \text{if} & SAS_{score\geq 5} \end{array} \end{array}$$ (3)

The four sub-scores in Fig 1 are the four components of *SAS*_*score*_, which have to be added together. For example, suppose we have a male patient with *BMI* = 39, *NC* = 46, *SysBP* = 140, and *ESS* = 8. Using a printed copy of Fig 1, one could note the approximate values on the y-axis that correspond to each measure found on the x-axis. As such, the sub-score for *BMI*, corresponding to x-axis value 39, is the y-axis value of 1.3; the sub-score for *NC* is 1.1; the sub-score for *SysBP* is 1; the sub-score for *ESS* is 0.75. Adding these four values, we obtain *SAS*_*score*_ = 1.3 + 1.1 + 1 + 0.75 = 4.15, which corresponds to a high risk of OSAS, according to Eq 3.

**Fig 2:**
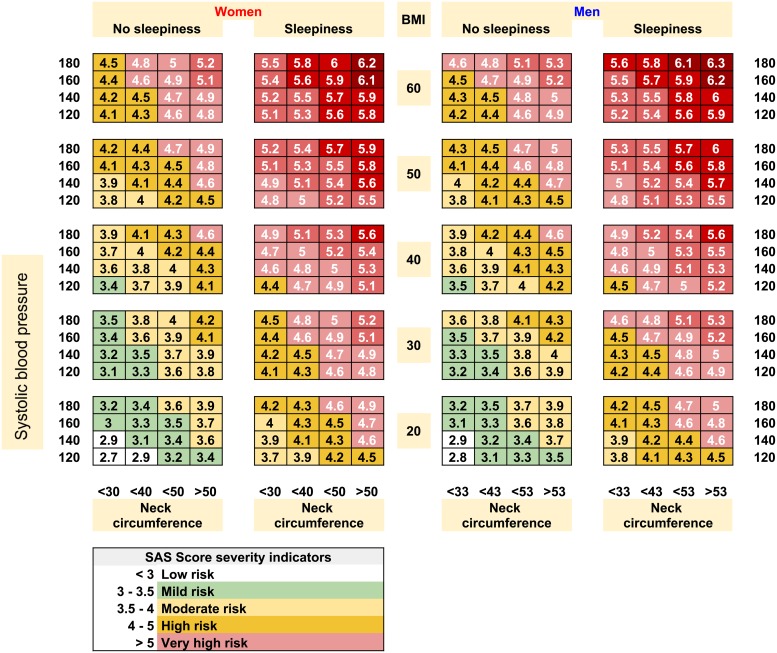
The sleep apnea syndrome severity scorecard that provides an approximation for *SAS*_*score*_, based on patient sex, BMI, neck circumference, systolic and diastolic blood pressure, and daytime sleepiness.

To further enhance the usability of *SAS*_*score*_, we propose an OSAS severity scorecard as presented in Fig 2. The scorecard fosters quick diagnosis for any new patient, which can be a very helpful tool for family doctors, or even for population-wide self assessment.

## Results

The clinical parameters, demographic and anthropometric data for the 2595 participants in our WestRo study cohort are shown in Table 1, alongside standard deviation (SD) or percentage (%) values.

**Table 1:** Demographic, anthropometric data, and clinical parameters of the WestRo patient cohort (*N* = 2595).

	Mean/*n*	SD/%*N*
Age (years)	52.2	±13.25
Gender (male)	1795	69.2%
Body-mass index (*kg*/*m*^2^)	33.73	±7.11
Obesity (*BMI* > 30)	1737	66.9%
Neck circumference (*cm*)	43.15	±5.25
Abdominal circumference (*cm*)	115.76	±17.07
Hypertension	1752	67.5%
Snoring	2024	78.0%
AHIs ≥ 30/*h*	1671	64.4%
Mean AHI	43.54	±26.57
Obstructive apneas	23.91	±23.86
Central apneas	3.06	±5.42
Mixed apneas	3.39	±6.38
Hypopnea	13.40	±9.91
Epworth sleepiness score ESS (0-24)	11.16	±5.40
Sleepiness (*ESS* ≥ 11)	1712	65.9%
STOP-Bang score ≥ 3	2404	92.6%
NoSAS score ≥ 8	2157	83.1%

The values in Table 1 represent means (accompanied by standard deviation *σ*), or counts *n* (accompanied by percentage % of total *N*). Hypertension is defined as systolic blood pressure of ≥ 140 mmHg or diastolic blood pressure ≥ 90 mmHg.

Our study group consists mainly of male patients (69.2%) with increased clinical signs of severe OSAS (64.4% have AHI > 30/*h*). As such, because our cohort mostly includes sick patients, the overall sensitivity of our results is higher and the measured specificity is lower than one would expect in a random population.

The performance results of our score is presented in Table 2. The prevalences of OSAS in the cohort, as can be defined by adopting different AHI cut-off values (the exact AHI values were measured under medical supervision) are as follows: 2519 (97.1%) for *AHI* ≥ 5, 2390 (92.1%) for *AHI* ≥ 10, 2238 (86.2%) for *AHI* ≥ 15, 2033 (78.3%) for *AHI* ≥ 20, 1671 (64.4%) for *AHI* ≥ 30, and 1093 (42.1%) for *AHI* ≥ 45. Table 2 provides the performance comparisons for the *AHI* = 30 cut-off.

**Table 2:** Performance of STOP-Bang, NoSAS, and *SAS*_*score*_ in the WestRo cohort (*N* = 2595) when *AHI* ≥ 30 events/h is considered the diagnosis criteria.

	Prevalence	AUC	Sensitivity	Specificity	PPV	NPV
STOP-Bang	2404 (92.6%)	0.69 (0.66-0.73)	0.968	0.149	0.673	0.723
NoSAS	2157 (83.1%)	0.66 (0.63-0.68)	0.901	0.294	0.698	0.621
*SAS*_*score*_	1977 (76.2%)	0.73 (0.71-0.75)	0.829	0.359	0.701	0.537

The data within parentheses (from the ‘AUC’ column) represent 95% confidence intervals. AUC = area under the curve. PPV = positive predictive value. NPV = negative predictive value.

Overall, we notice that the prevalence according to the *SAS*_*score*_ (76.2%) is the closest to the real one (64.4%)—as obtained after rigorous polysomnography—and the AUC has the highest value (0.73) for *SAS*_*score*_. In terms of sensitivity, *SAS*_*score*_ performs marginally weaker (0.829), yet it offers the best specificity among the three scores (0.359). These results mean that *SAS*_*score*_ obtains a specificity that is 140.9% higher than that of STOP-Bang.

In Table 3 we provide the values for true/false positives/negatives obtained by applying the three scores on the WestRo dataset. Again, we notice that *SAS*_*score*_ attains a better patient filtering. For example, when analysing the true negative rate of STOP-Bang, we estimate that the diagnosis finds 138 healthy patients out of 924 (14.9%), while *SAS*_*score*_ finds 331 healthy patients out of the 924 healthy ones (35.9%). Moreover, in terms of false positive rate, STOP-Bang falsely diagnoses 786 patients (85.1% of the healthy population); *SAS*_*score*_ falsely predicts only 591 (63.9%) patients.

**Table 3:** Correct classification and missed diagnosis for the WestRo cohort (*N* = 2595).

	STOP-Bang		NoSAS		SAS_*score*_	
	Positive	Negative	Positive	Negative	Positive	Negative
AHI ≥ 30 events/h (positive)	1618 (62%)	53 (2%)	1505 (58%)	166 (6%)	1386 (53%)	285 (11%)
AHI < 30 events/h (negative)	786 (30%)	138 (5%)	652 (25%)	272 (10%)	591 (23%)	331 (13%)

Data are n (%). AHI = apnoea-hypopnoea index. Prevalence of OSAS by polysomnography is 64.4%.

We also present a cross-validation analysis of our *SAS*_*score*_, using an independent dataset, which we refer to as the *CPAP* cohort (provided in S2 Dataset). This independent dataset includes relevant data for *N*_*CPAP*_ = 231 patients, gathered during autumn 2013, by following the same procedure as our *WestRo* cohort (with *N*_*WestRo*_ = 2595 patients). The *CPAP* cohort data was gathered in a sleep laboratory in Timisoara, Romania, where overnight CPAP treatment was performed. Considering only the data that is relevant to our current study, we are able to measure *SAS*_*score*_ for each patient, and then determine the corresponding AUC, sensitivity and specificity for the entire *CPAP* dataset. In Table 4 we present the anthropometric data of the *CPAP* cohort. The statistical results for *SAS*_*score*_ in the *CPAP* cohort are very similar to the results obtained for *WestRo*: OSAS prevalence of 171 (74.0%) where real prevalence is 157 (67.9%); AUC of 0.70 (CI 0.68-0.72), sensitivity of 0.803, specificity of 0.392, PPV of 0.737, and NPV of 0.483. Indeed, the cross-validation proves that *SAS*_*score*_ is an accurate and robust predictor of OSAS.

**Table 4:** Demographic, anthropometric data, and clinical parameters of the CPAP patient cohort (*N* = 231).

	Mean/*n*	SD/%*N*
Age (years)	52.01	±13.61
Gender (male)	167	72.3%
Body-mass index (*kg*/*m*^2^)	32.72	±7.39
Obesity (*BMI* > 30)	154	66.7%
Neck circumference (*cm*)	42.64	±5.11
Hypertension	150	64.9%
AHIs ≥ 30/*h*	157	67.9%
Mean AHI	44.39	±26.13
Epworth sleepiness score ESS (0-24)	11.38	±5.19
Sleepiness (*ESS* ≥ 11)	127	54.9%

The values in Table 4 represent means (accompanied by standard deviation *σ*), or counts *n* (accompanied by percentage % of total *N*). Hypertension is defined as systolic blood pressure of ≥ 140 mmHg or diastolic blood pressure ≥ 90 mmHg.

**Fig 3:**
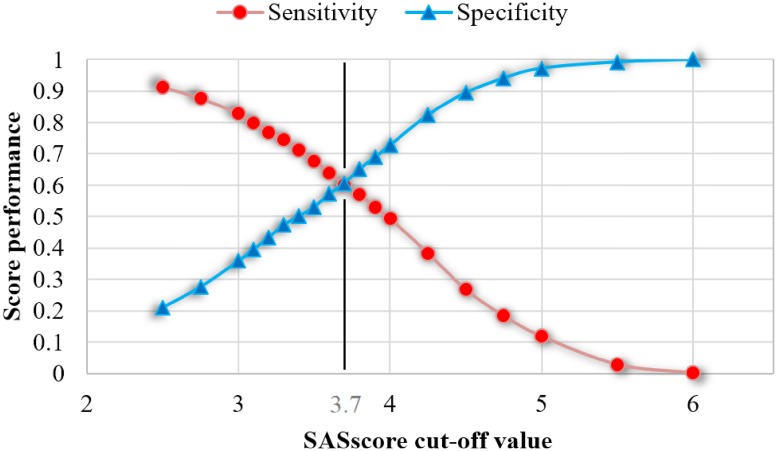
State space exploration of cut-off values for the *SAS*_*score*_ to observe the sensitivity-specificity interplay. The point of intersection between sensitivity and specificity gives the cut-off value of 3.7.

All the discussed results are obtained by considering the cut-off value of 3 for our *SAS*_*score*_. According to Eq 3, this value of 3 coincides with the threshold between *Low* (no) risk and *Mild* risk of OSAS. To further explore the consequences of modifying the threshold value, we represent sensitivity and specificity that are obtained by increasing the value of *SAS*_*score*_ cut-off, starting from 2.5 up to 6 (see Fig 3).

By changing the value of *SAS*_*score*_ cut-off we can simply alter the diagnosis outcome—from a very permissive score (i.e., low cut-off translates to high sensitivity, similar to STOP-Bang), to a very strict one (i.e., high cut-off translates to high specificity). As shown in Fig 3, it is not possible to keep high levels for both sensitivity and specificity, therefore we try to find a balance between the two. As our study goal is to attain a higher specificity, we adopt the *SAS*_*score*_ cut-off value of 3.7 according to the empirical results from Fig 3. In other words, a patient is considered at risk of OSAS, if his or her *SAS*_*score*_ ≥ 3.7.

As such, depending on the actual clinical context, the cut-off value may be considered too small for preoperative diagnosis (which we further refer to as case A), or too high for population monitoring (case B). In case A, one could use a cut-off value of 2.5-2.75, thus obtaining a score that is similar to NoSAS in terms of sensitivity (0.913-0.876) and specificity (0.211-0.277). In case B, one could use a cut-off value of 4-4.5, to obtain a lower sensitivity (0.494-0.268), but a significantly improved specificity (0.727-0.894).

**Fig 4:**
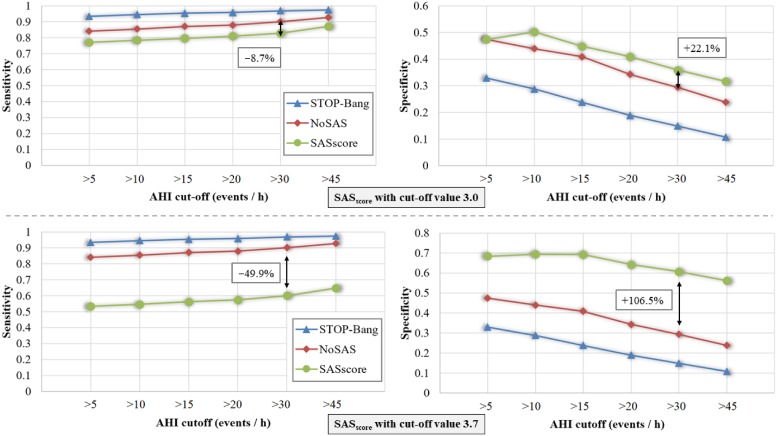
*SAS*_*score*_ performance compared with STOP-Bang and NoSAS scores in terms of sensitivity and specificity over different AHI cut-off values. The upper panel corresponds to a *SAS*_*score*_ with cut-off value 3.0, while the lower panel corresponds to the empirically determined optimal cut-off value of 3.7.

For better understanding the impact of the cut-off value, we compare *SAS*_*score*_ with cut-offs 3 and 3.7 against both STOP-Bang and NoSAS; Fig 4 plots the performances of the three scores accordingly. Note that the reference results for STOP-Bang and NoSAS remain the same in the two panels of Fig 4, because they are independent of our score’s customization.

## Discussion

Our results show that, using patient measurements that are easily available in primary care practice, the customizable *SAS*_*score*_ allows for reliable determination of clinically significant OSAS, with a high and adjustable specificity, ranging from 0.359 to 0.607. Compared with existing state of the art screening scores, such as STOP-Bang (0.149 specificity) and NoSAS (0.294 specificity), *SAS*_*score*_ is indeed the most appropriate for monitoring large populations.

The task of developing an ideal OSAS screening score is cumbersome, because of the several possible application contexts [4]. For instance, in a *clinical context* involving preoperative phases, a score should mainly have a high sensitivity to avoid the potentially catastrophic consequences of false-negative results. Conversely, in a *primary care context*, the score should additionally be specific enough to avoid referral of low-risk patients for costly and time-consuming polysomnography. In a *population-wide context*, including family doctors and self-assessment of SDB, the score should **mainly focus on specificity** in order to avoid high false-positive rates. Moreover, specificity is especially important for low prevalence populations [5, 6, 35]. Currently, there exist relatively good solutions for the first two exemplified contexts, namely the Berlin, STOP-Bang, and NoSAS scores. However, an efficient tool for the third mentioned context (i.e. population-wide) is yet to be developed. As suggested by its higher AUC and correct classification proportion (with respect to the other scores), our *SAS*_*score*_ has the potential of representing a better compromise between sensitivity and specificity, allowing clinically significant SDB to be reliably ruled out, without yielding too many unnecessary sleep investigations.

To achieve time efficiency, a screening score should entail a small number of measures. At the same time, such measures must be related to easily available and objective patient variables [36]. Similar to the NoSAS score, *SAS*_*score*_ uses anthropometric measures, such as BMI and systolic blood pressure (which are part of any standard clinical assessment), as well as neck circumference and Epworth score [34], which can be easily measured and assessed respectively. As *SAS*_*score*_ is based on a previously developed classifier [29] developed through means of machine learning and network science, the main aim of this paper is to develop an easy-to-apply, yet reliable score. Therefore, we try to limit the number of subjective variables, such as witnessed sleep apneas, or snoring severity and frequency, which require the subjective observation of a third party, thus affecting the robustness of the score.

Compared with the 8 questionnaire items required for STOP-Bang, the 9-11 questions of the Berlin questionnaire, or the 5 items of the NoSAs score, this new version (i.e. not computer-based) of *SAS*_*score*_ only requires 4 items, thus being very appropriate for clinical practice. Indeed, *SAS*_*score*_ may be easily computed by hand, with a tablet, or a smartphone. We have also developed a website (sasscore.appspot.com) which produces the calculations on demand, as well as a smartphone application, currently available on the Android platform (Morpheus: Sleep Apnea Syndrome app on Google Play: https://play.google.com/store/apps/details?id=aerscore.topindustries.aerscore&hl=en).

### *SAS*_*score*_ assessment limitations

The robustness of *SAS*_*score*_ relies on the accuracy of measuring the involved parameters, such as BMI, systolic blood pressure, ESS, etc. In some cases, measuring these parameters may lead to inaccurate results, thus affecting our score’s reliability. At the same time, the accuracy of parameter measurements depends on the context in which the assessment is made: self-assessment at home or medical assessment in primary care units.

BMI varies over time, mostly because of weight variation. In our *WestRo* cohort, all the patients were measured under medical supervision (at every visit) with a standardized and validated scale for weight, height (thus rendering a reliable BMI), and for neck circumference. Indeed, these anthropometric measurements can be performed reliably in primary care units.

For our *WestRo* dataset, systolic and diastolic blood pressure were measured with a standard blood pressure monitor under medical supervision. The diagnostic of systemic high blood pressure was made by considering blood pressure measurements as well as patient’s medical history. However, primary care doctors should be aware of potential problems such as the white coat hypertension; if they suspect such cases, medical doctors can decide on future recurrent assessments.

ESS alone has considerable limitations, due to its low predictive value for patients with subjective excessive daytime sleepiness. However, ESS is still the most used sleepiness score in clinical practice worldwide; for better efficiency, as we did for our *SAS*_*score*_, it is usually combined with other objective measurements [37].

Both self-assessment and primary care assessment have advantages and disadvantages in terms of reliably measuring the relevant parameters. However, we recommend the more dependable alternative, namely assessing *SAS*_*score*_ in primary care. Our score can be determined by self-assessment also, but merely as an indicator which is intended to make people aware of OSAS and its consequences; if the *SAS*_*score*_ value measured by self-assessment would suggest a high risk, then we recommend referring to a primary care unit.

### Potential *SAS*_*score*_ applications

Our score can be a useful tool for OSAS/SDB screening in large population categories such as professional drivers, because, from January 2016, the new 2014/85/EU directive [38] targeting professional drivers is recommended across the entire European Union (Commission Directive 2014/85/EU of 1 July 2014 amending Directive 2006/126/EC—European Parliament and the Council on driving licences).

To this end, as presented in the previous subsection, we recommend that the score assessment be performed by primary care physicians, to ensure the accuracy of parameter measurements. If the *SAS*_*score*_ determined by the primary care physician indicates a low risk (according to Eq 3), then the subject can be ruled out from the suspicion of OSAS. If *SAS*_*score*_ indicates a very high risk, then the subject is diagnosed with OSAS; in some cases, simpler devices such as portable respiratory polygraphs (for home usage) may be employed to confirm the diagnosis. However, low and very high risk are the clear-cut cases. If the physicians are dealing with borderline cases (i.e. mild, moderate, and high risk), then full fledged hospital polysomnography is recommended to provide a more accurate assessment.

## Conclusion

In this paper we present the optimized *SAS*_*score*_, which proves to be more efficient than existing scores such as STOP-Bang or NoSAS when monitoring OSAS in large populations. In comparison with NoSAS, *SAS*_*score*_ provides only marginally lower sensitivity, but achieves a much desired higher specificity. Furthermore, *SAS*_*score*_’s diagnosis cut-off value can be customized to increase either sensitivity or specificity, while maintaining the AUC value in an optimal balance. The applicability of our proposed tool is wide, and represents a timely advancement in the field of OSAS monitoring.

## Supporting information

S1 Table AHI and relevant risk factor parameters distribution. The distribution of AHI values and relevant risk factor parameters distribution in the *WestRo*—Western Romania dataset (*N* = 2595 subjects), given as average values and standard deviation.
(PDF)

S1 Fig Anthropometric measurements distributions. Distributions of AHI, body mass index (BMI), age, neck circumference (Neck), systolic blood pressure (SysBP), and Epworth sleepiness score (ESS) in the *WestRo* cohort. The results show that, given that the database contains subjects from a well-delimited geographical area which were randomly gathered, the main parameters are normally distributed.
(EPS)

S1 Dataset *WestRo* dataset. (XLSX)

S2 Dataset *CPAP* dataset. (XLSX)
