# Supplementary material for: SAS score: Targeting high-specificity for efficient population-wide monitoring of obstructive sleep apnea
Source: PLoS One. 2018 Sep 5;13(9):e0202042. doi: 10.1371/journal.pone.0202042 (PMC6124708; doi:10.1371/journal.pone.0202042)
Supplement: S1 Table — The distribution of AHI values and relevant risk factor parameters distribution in the WestRo—Western Romania dataset (N = 2595 subjects), given as average values and standard deviation. (PDF) [file pone.0202042.s001.pdf]

**Parameter**	**Average**	**Std. dev.**
AHI	43.91	26.35
BMI	33.76	7.16
Age	52.19	13.27
NC	43.19	5.25
SysBP	135.45	17.21
ESS	11.16	5.41
